# Chinese validation and clinical application of the tinnitus functional index

**DOI:** 10.1186/s12955-020-01514-w

**Published:** 2020-08-06

**Authors:** Xianren Wang, Ruyan Zeng, Huiwen Zhuang, Qiyang Sun, Zijun Yang, Cangjian Sun, Guanxia Xiong

**Affiliations:** 1 Department of Otorhinolaryngology, The First Affiliated Hospital, Sun Yat-Sen University, Guangzhou, 510080 People’s Republic of China; 2 Institute of Otorhinolaryngology, Sun Yat-Sen University, Guangzhou, 510080 People’s Republic of China

**Keywords:** China, Reliability and validity, Tinnitus, Index

## Abstract

**Objective:**

The Tinnitus Functional Index (TFI) is a new diagnostic measure of the functional impact of tinnitus that is also a sensitive measure of treatment-related changes. However, the TFI has not been translated into Chinese and fully validated in China. The aim of the present study was to evaluate the validity of a Chinese version of the TFI as a diagnostic measure of tinnitus severity in a sample of Chinese patients and to verify the value of its clinical application in China.

**Design:**

A sample of 206 patients whose primary complaint was tinnitus was used to analyze the reliability and validity of the TFI. In addition, patients were asked to fill out the Tinnitus Handicap Inventory (THI) and the Center for Epidemiologic Studies-Depression Scale (CES-D), the Beck Anxiety Inventory (BAI), and the Satisfaction With Life Scale (SWLS) to compare TFI with their association. The internal consistency of the TFI was assessed with Cronbach’s alpha coefficient. The factor structure of the TFI was assessed by Exploratory Factor Analysis (EFA). The extracted factors were compared to those of the original TFI scale.

**Results:**

The reliability of the Chinese version of the TFI (Cronbach’ s α = .969) showed high internal consistency. The exploratory factor analysis (EFA) of the TFI showed that six factors with one main factor could be extracted instead of eight factors as described in the original version. Nevertheless, relations to the original eight subscales could be demonstrated. A high correlation between the TFI and the THI (*r* = .865, *p* < 0.01) and lower correlations between the TFI and the CES-D (*r* = .334, *p* < 0.01), BAI (*r* = .559, *p* < 0.01), and SWLS (*r* = − 0.324, *p* < 0.01) confirmed the satisfactory convergent and discriminant validity of the TFI.

**Conclusion:**

After translated and validated a Chinese version of the TFI and found that the TFI had high reliability and validity, which means both instruments are reliable instruments to assess the severity of tinnitus in clinical applications in China.

## Introduction

Tinnitus is a subjective sensation in the ear without a corresponding external sound source or external stimulus [1]. Epidemiology study estimate that 10–15% of the adult population experiences chronic tinnitus [2]. However, there currently is no large-scale epidemiological survey of tinnitus in China. Some researchers have conservatively estimated that 10.4% of people in south China have experienced tinnitus, of which 5% have sought medical treatment, and 2% have been seriously affected by it with respect to life, sleep, work, and social activities [3]. In addition, 0.5% of patients with tinnitus in this population have a disability because of its severity [3]. The mental and psychological problems caused by tinnitus are becoming increasingly prominent, and more attention needs to be paid to the association between tinnitus severity and anxiety and depression [4].

As tinnitus is a subjective feeling, there is no objective method to measure its severity or assess the effects of treatment for tinnitus [5]. Therefore, it has been necessary to use scales or other types of questionnaires to evaluate the severity of tinnitus and patients’ responses to treatment quantitatively.

Several questionnaires have been developed over the years to evaluate the severity of tinnitus, including the Tinnitus Handicap Inventory (THI) [6], the Tinnitus Handicap Questionnaire (THQ) [7], the Tinnitus Activity Questionnaire (TAQ) [8], the Tinnitus Reaction Questionnaire (TRQ) [9], the Tinnitus Questionnaire (TQ) [10], and the Tinnitus Functional Index (TFI) [11]. Most of Chinese language tinnitus questionnaires that are in use have not been translated or validated according to accepted scientific methodology [12]. In China, the THI scale is widely used in China after being translated and verified by Qiulan Shi [13]. It is a scale commonly used in China to assess the severity of tinnitus. Therefore, in order to verify the assessment of tinnitus severity on the TFI scale, it can be verified with the THI scale. Those that have been statistically validated, such as the THI, TAQ, and THQ [14], were not developed specifically to detect changes in tinnitus-related treatment. The TFI, on the other hand, was specifically developed to be both a diagnostic measure of the functional impact of tinnitus and to be a sensitive measure of treatment-related changes [11]. It has been translated to more than 10 languages and has demonstrated convergent validity [15]. TFI consists of eight sub-scales, which are used to quantitatively evaluate the degree of patients’ tinnitus effects in the dimensions of Intrusive, Sense of control, Cognitive, Sleep, Auditory, Relaxation, Quality of life, and Emotional. Thus the evaluation scale in view of the wider range, and excellent psychometric properties [11, 16, 17]. Therefore, the primary goal of this study was to produce a cross-culturally valid Chinese-language version of the TFI and to report its psychometric properties, in order to provide clinicians and researchers who work within the tinnitus field in China with an alternative instrument to those already in use.

## Materials and methods

### Research participants

A total of 206 outpatients in the First Affiliated Hospital of Sun Yat-sen University were recruited for the study, 175 participants were included from May to September 2018 and the others were recruited from March to May 2020. The inclusion criteria were patients who had tinnitus for longer than 6 months, normal intelligence, no communication barriers, the ability to understand the content of the scales, and the ability to answer the questions. The exclusion criteria were objective tinnitus or a history of suppurative otitis media. The study participants were 106 males and 100 females between 18 and 65 years of age. Participation was voluntary, and all the participants gave oral consent before completing the scales; the data were stored and analyzed anonymously.

### Translation and cross-cultural adaptation

The original version of the TFI was independently translated into Chinese by the two authors, both of whom are native Chinese speakers with advanced skills in the English language and expertise in tinnitus. A third forward translation was made by a bilingual expert in healthcare questionnaires with no prior knowledge of the TFI. The three translations were then compared and synthesized into the first prototype of the TFI-CN. Then, two back-translations were performed by a bilingual medical scientist and a professional translator. The back-translations were subsequently compared to the original TFI by an expert panel consisting of the authors, the translators, and three colleagues from tinnitus clinics. All discussions, comments, and the final revision were documented [18].

### Research instruments

#### Tinnitus handicap inventory (THI)

The THI consists of 25 questions that are answered “Yes,” “Sometimes,” and “None.” And the scale is divided into three subscales: functional, catastrophic, and emotional. The scale’s total possible score is 100 points: 4 points for “Yes,” 2 points for “Sometimes,” and 0 points for “None.” Newman et al. divided tinnitus disability into four levels. The first level = 0 to 16 points (no disability); the second level = 18 to 36 points (mild disability); the third level = 38 to 56 points (moderate disability); and the fourth level = 58 to 100 points (severe disability).

#### Tinnitus functional index (TFI)

The TFI consists of 25 items that are divided into eight sub-scales of 3–4 items that measure the severity of different problems related to tinnitus. The scale is divided into 8 sub-scales (Intrusive, Sense of control, Cognitive, Sleep, Auditory, Relaxation, Quality of life, and Emotional) to deal with different areas of tinnitus severity. Each item is rated on a 0 to 10 scale (0 = never or not at all; 10 = almost always or extremely), with the raw scores on each subscale multiplied by 10 to yield subscale scores of 0 to 100. The total score is the average of the eight sub-scale scores.

#### Center for Epidemiologic Studies-Depression Scale (CES-D)

The CES-D, which was developed by Radloff at the National Institute of Mental Health in 1977, can be used as a clinical examination to assess the severity of depressive symptoms [15, 16]. The scale consists of 20 items, with each item measuring a symptom that respondents may have experienced during the past week. The response options are: “less than one day” (“no or not”); “1 to 2 days” (“rare”); “3 to 4 days” (“often”); and “5 to 7 days” (“almost always”). The main CES-D measure is its total score. A total score ≤ 15 indicates no depressive symptoms, 16 to 19 points indicates the respondent may have depressive symptoms, and ≥ 20 points indicates the respondent must have depressive symptoms.

#### Satisfaction with life scale (SWLS)

The SWLS consists of 5 statements that are scored from 1 (strongly disagree) to 7 (strongly agree), and summed to form a total score. Higher scores indicate greater life satisfaction [19].

#### Beck anxiety inventory (BAI)

The BAI is a self-report measure of anxiety that consists of 21 symptoms of anxiety. Respondents are asked to rate the degree to which they have been bothered or annoyed by each symptom during the past month on a 4-point scale: “not at all” = 1; “mildly, no major annoyance” = 2; “moderately, feels uncomfortable but still tolerable” = 3; and “severe, can barely endure” = 4 [20].

### Statistical analysis

#### Processing of missing data

All the participants were given oral instructions about how to complete the scales and were asked not to omit any items or mark more than one answer for each item. According to the instructions of the authors of the original scales for computing the subscale and total scores in cases where the scales were incomplete. If two or more questions are not answered, the scale was considered invalid and rejected. The missing data were estimated using the multiple imputation [21] .

#### Pearson’s correlations

The convergent validity of the TFI was compared with THI, and the discriminant validity of the TFI with the CES-D, SWLS, and BAI were analyzed using Pearson’s correlation coefficients. An *r* > .8 indicates a strong correlation between two variables; an *r* < .3 indicates a weak correlation between two variables.

#### Reliability analysis

The reliability of the Chinese versions of the TFI and THI were measured by Cronbach’s α coefficient for internal consistency; the internal consistency of the total scores of the TFI and THI scales and their individual items were tested. Cronbach’s α > .7 indicates the agreement between the items is good; Cronbach’s α < .5 indicates items need to be eliminated.

#### Validity analysis

Bartlett’s Test of Sphericity and the Kaiser-Meyer-Olkin (KMO) test were conducted to evaluate the appropriateness of performing factor analysis. The factor analysis tested the eigenvalues of the 25 items on the scale, identified the items with eigenvalues greater than 1, or the Jolliffe’s criterion has a suggested cut-off for eigenvalues greater than 0.7 [22], and calculated the cumulative variance to extract the common factors in the scale.. The significance level was set at *p* ≤ 0.05 (two sided), unless otherwise specified. The factor matrix was rotated to determine the items corresponding to each dimension, and the items with the highest commonality on the scale were evaluated. Factor analysis was carried out using the oblique rotation [16, 23, 24]. The McDonald’s omega, including hierarchical subscale and omega total, are also used to further evaluate the dimensionality of the scale [25]. The above analyses were computed with the latest R (4.0.2) software (The R Foundation for Statistical Computing, Vienna, Austria) with psych package and IBM SPSS version 22 (International Business Machines Corporation, Armonk, NY, USA).

## Results

### Study participants

A total of 206 outpatients with tinnitus were included in this study. The sample consisted of 106 males and 100 females between the ages 18 and 65 years-old; mean = 38.1 (±12.4 SD) years-old. The mean duration of tinnitus was 10.6 months (±12.5 SD, range = 6–720 months). All the participants received and completed the Chinese version of the THI and TFI, for a response rate of 100%, as shown in Tables 1 and 2; 161 of the participants also completed the CES-D, BAI, and SWLS.

**Table 1:** The score of and TFI (*n* = 206)

Scale	Average	Standard deviation
TFI	33.14	22.75
Intrusive	48.46	26.82
Sense of control	38.66	23.91
Cognitive	29.22	26.49
Sleep	34.14	31.94
Auditory	23.82	27.16
Relaxation	36.68	30.17
Quality of life	22.44	25.69
Emotional	35.29	28.61

**Table 2:** Tinnitus severity rating of TFI (*n* = 206)

Scale	Severity	Patient n	Percent %
TFI	Not a problem	68	33
	A small problem	44	21
	A moderate problem	57	28
	A big problem	28	14
	A very big problem	9	4

### Correlation and discriminant validity between the THI, TFI and other scales

There was a strong positive correlation between the THI and TFI: *r* = .865, *p*<0.01. In addition to the negative correlation between SWLS and TFI scales, the other two psychological scales were positively correlated with TFI scales, and had a moderate correlation, all *p*<0.01, as shown in Table 3.

**Table 3:** Correlations between the TFI and other measures used in the adaptation procedure

	CES-D^c^	BAI^c^	SWLS^c^
TFI^b^	0.334^a^	0.559^a^	−0.324^a^

^a^*P* < 0.01; *TFI* Tinnitus Functional Index, *CES-D* Center for Epidemiological Studies Depression, *SWLS* Satisfaction With Life Scale, *BAI* Beck Anxiety Inventory. ^b^: *n* = 206; ^c^: *n* = 161

### Reliability of the Chinese versions of the TFI

Cronbach’s α (0.969), omega hierarchical (0.87) and omega total (0.99) for the Chinese TFI, which indicates the internal consistency of all the items in the scale was good (see Table 4). The correlations between the items and the total score (item-total correlations) ranged from .573 to .837(detail see Table 5).

**Table 4:** Cronbach’s α analysis of each subscale of TFI (*n* = 206)

Scale	Cronbach’s α
TFI	0.968
Intrusive	0.849
Sense of control	0.836
Cognitive	0.937
Sleep	0.945
Auditory	0.942
Relaxation	0.939
Quality of life	0.919
Emotional	0.919

*TFI* Tinnitus Functional Index

**Table 5:** Corrected item total correlation for all items of the TFI (*n* = 206)

Item	Corrected item-total correlation
I1	0.573
I2	0.729
I3	0.695
SC1	0.779
SC2	0.689
SC3	0.721
C1	0.837
C2	0.758
C3	0.756
SL1	0.673
SL2	0.728
SL3	0.670
A1	0.675
A2	0.687
A3	0.719
R1	0.742
R2	0.778
R3	0.748
Q1	0.792
Q2	0.806
Q3	0.770
Q4	0.768
E1	0.785
E2	0.812
E3	0.710

*I* Intrusive, *SC* Sense of control, *C* Cognitive, *SL* Sleep, *A* Auditory, *R* Relaxation, *Q* Quality of life, *E* Emotional

### Validity of the Chinese TFI using exploratory factor analysis

**Fig. 1:**
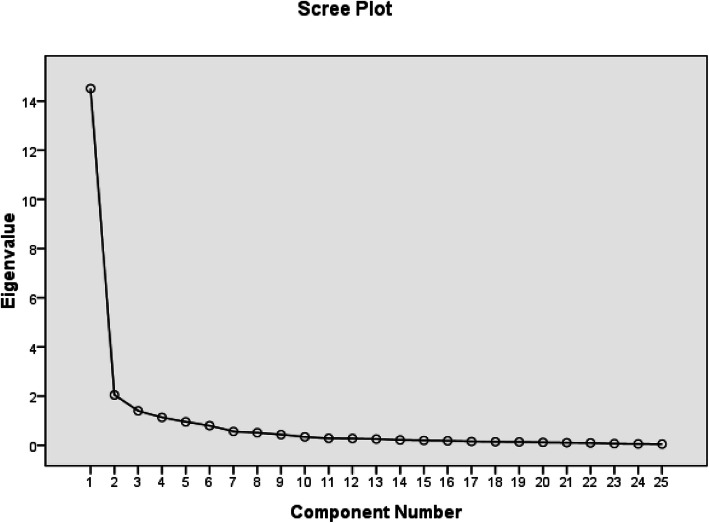
Scree Plot of principal component analysis with oblique rotation. The scree plot showed a very sharp decline after the first factor. A virtually drawn horizontal and vertical line starting from each end of the curve indicated only one factor point above the point of inflexion

The Kaiser-Meyer-Olkin (KMO) Measure of Sampling Adequacy and Bartlett’s Test of Sphericity were assessed to assure that the data were eligible for exploratory factor analysis. The Bartlett’s test of Sphericity was significant (< 0.001), indicating that the items of the TFI were correlated. The KMO value was 0.936, which means the partial correlations were weak, indicating that the scale was suitable for factor analysis. Principal component analysis with Promax rotation was used to analyze the scale and the resulting communalities ranged from 0.64 to 0.94. The number of components of the TFI was defined based on the eigenvalues. The scree plot showed a very sharp decline after the first factor (Fig. 1). and virtually drawn horizontal and vertical lines starting from each end of the curve indicated that only one factor point was above the point of inflexion. However, by applying Jolliffe’s criterion, a six-factor structure could be retained (Table 6) and these six factors explained 83.35% of the total variance. With reference to the original TFI’s eight factors, a principal component analysis with promax rotation and eight fixed factors was performed (Table 7). Although the eigenvalues of the last factors were smaller than 0.7, there were correlations between the items and their factors. Only Factor 8, standing for quality of life, was represented by only one item, Q4. Items Q1 and Q3 were also expected to correlate with this factor but did not. Rather, both Items correlated strongly with Factor 1 (intrusiveness). Q2 loaded on the second factor, standing for relaxation. as shown in Table 7. These results indicated that the instrument was structurally efficient.

**Table 6:** Principal component analysis with Promax rotation

Item	Factor					
	1	2	3	4	5	6
A3	0.860					
A2	0.854					
A1	0.770					
Q1	0.680	0.422				
Q3	0.622	0.396				
Q4	0.549					
R3		0.771				
R1		0.757				
R2		0.750				
Q2	0.470	0.596				
SL2			0.857			
SL1			0.856			
SL3			0.764			
I3				0.722		
I1				0.722		
I2				0.597		
SC2			0.355	0.561		
SC3				0.543		
C2					0.746	
C3	0.367				0.711	
C1			0.352		0.623	
SC1			0.383		0.460	
E1		0.355				0.698
E2						0.680
E3						0.597

**Table 7:** Rotated factor loading matrices of the predefined-factor models with fixed eight factors

Item	Factor							
	1	2	3	4	5	6	7	8
A2	1.041							
A3	1.017							
A1	0.962							
Q1	0.515							
Q3	0.375							
R1		1.038						
R3		0.988						
R2		0.961						
Q2		0.489						
SL3			0.985					
SL1			0.981					
SL2			0.911					
C2				1.050				
C3				1.047				
C1				0.737				
E1					0.915			
E3					0.888			0.470
E2					0.833			
I1						1.084		
I3						0.861		
SC2							0.677	
SC3				0.352			0.544	
I2							0.443	
Q4	0.368							0.573
SC1				0.362				0.569

*I* Intrusive, *SC* Sense of control, *C* Cognitive, *SL* Sleep, *A* Auditory, *R* Relaxation, *Q* Quality of life, *E* Emotional

### Factorial validity

**Fig. 2:**
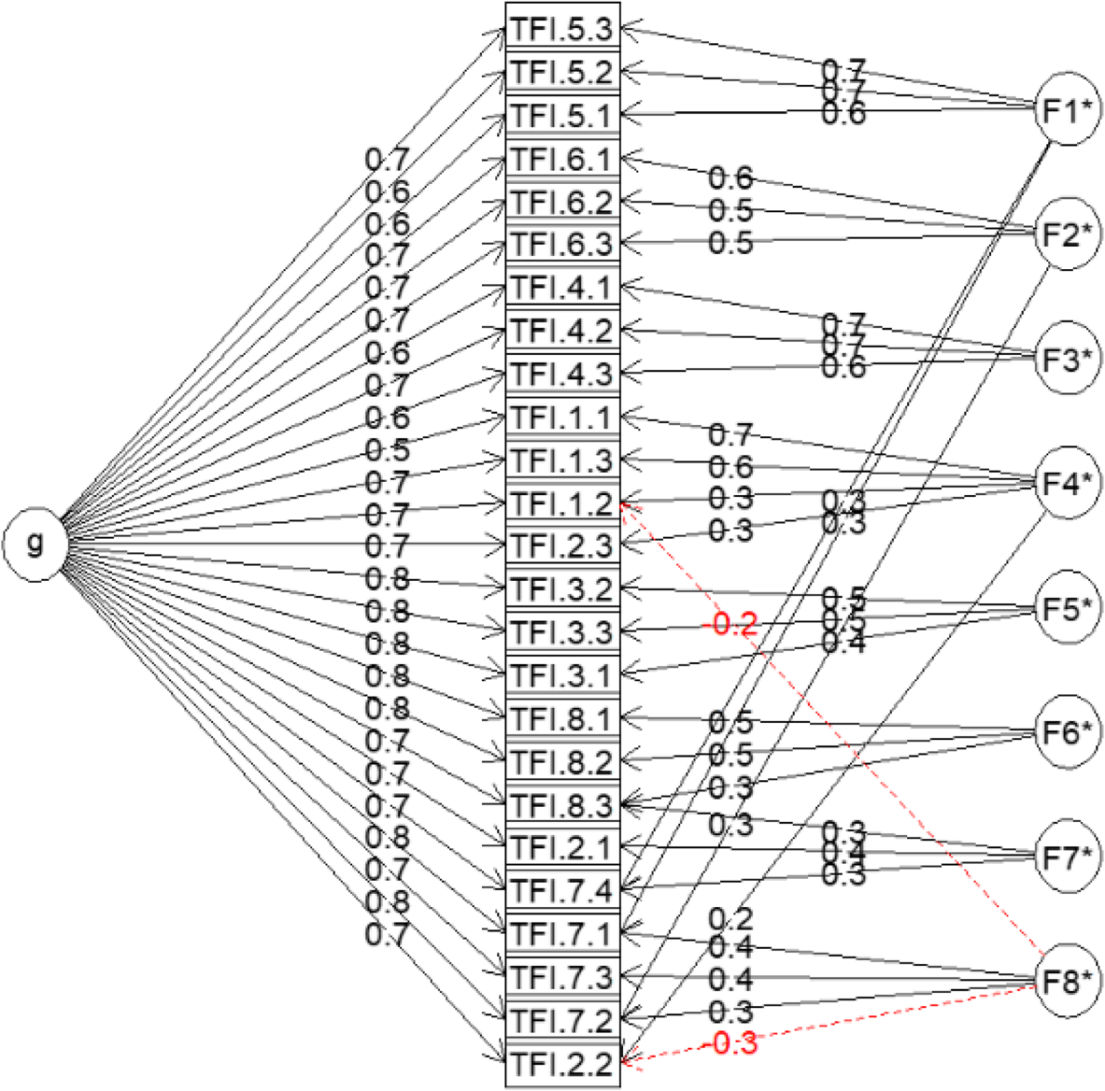
Estimated model for the 25-item TFI Scale with eight-factor structure. TFI.1 represent Intrusive; TFI.2represent Sense of control; TFI.3 represent Cognitive; TFI.4 represent Sleep; TFI.5 represent Auditory; TFI.6 represent Relaxation; TFI.7 represent Quality of life; TFI.8 represent Emotional

To reconfirm the EFA results, the McDonald omega hierarchical subscale analyses were further evaluated. The McDonald omega results indicated that the TFI-CN possesses an eight-factor structure with relatively large omegaHS values [25] (ω_hs.I_ = 0.57; ω_hs.SC_ = 0.64; ω_hs.C_ = 0.68; ω_hs.SL_ = 0.48; ω_hs.A_ = 0.47; ω_hs.R_ = 0.60; ω_hs.Q_ = 0.75; ω_hs.E_ = 0.67; I, Intrusive; SC, Sense of control; C, Cognitive; SL, Sleep; A, Auditory; R, Relaxation; Q, Quality of life; E, Emotional), show as Fig. 2. The result indicated that that the 25-item TFI with an eight-factor structure possesses good factorial validity.

## Discussion

The TFI have been translated into many different languages since they were originally published and all the versions have demonstrated good reliability and validity [15, 17, 26, 27]. However, the TFI has not been translated into Chinese and validated in China. The present study found the Chinese versions of TFI had excellent internal consistency (Cronbach’s α of .969), similar to their Polish versions (Cronbach’s α of .960), and the original versions (Cronbach’s α of .97) [11, 24]. The TFI-CN’s convergent validity with the THI was high (*r* = .865) and comparable to the English version (*r* = .75). The positive correlations of the TFI-CN with the CES-D (*r* = .334, *p* < 0.01) and BAI (*r* = 0.559, *p* < 0.01) were stronger than the negative correlation between the TFI-CN and the SWLS (*r* = −.324, *p* < 0.01), all of which we interpret as satisfactory discriminant validity.

The Polish version of the TFI scale has an overall Cronbach’s α of .960, which is comparable to that of the Chinese version (Cronbach’s α = .969), but Cronbach’s α for the Chinese version is slightly higher. The item-total correlations of the Chinese TFI are .562–.848, whereas the item-total correlation of the Polish TFI are .83–.95. As the item-total correlations of these two versions of the TFI are .30 or more, this indicates the Chinese version of the TFI has good internal reliability.

This study used factor analysis to test the structural validity of the scales. Similar as the scree plot of the German version, TFI-CN also indicated only one dominant factor with a high eigenvalue of 14.51 and all following eigenvalues ≤2.1. However, using the Jolliffe’s criterion (eigenvalues > 0.7), six factors could be extracted. Other studies have also failed to reproduce the original eight factor structure. For example, in the validation of the Polish TFI [24] and German TFI [23], 5 factors were identified; and in the Swedish TFI [28] validation, 6 factors were extracted; in the Dutch version TFI 7 factors were found [16].

Of our extracted six factors when using the Jolliffe’s criterion (eigenvalues > 0.7), Factor 1 included two subscales of the original TFI consisting of Auditory and Quality of life. The validation of the Dutch TFI and German TFI, quality of life was loaded in one subscale with cognitive interference. This finding maybe can be explained Chinese of mandarin more focus on the hearing loss, hearing loss more effect on the quality of life. Factor 4 included two other subscales of the original TFI consisting of Intrusive and Sense of control. The version of German TFI has also the same result.

To verify the eight subscales of the original TFI, a factor analysis with eight fixed factors was performed. Despite the fact that the eigenvalues of the last two factors were smaller than 0.7, correlations between the items and their corresponding factors could be demonstrated. Only the last factor, Factor 8 (quality of life), was represented by only one item, Q4. The other items, Q1 and Q3, which were expected to correlate with this factor were strongly corelated with Factor 1 (auditory). Summary, the results of this study are similar to those from other studies validating the TFI in different languages. To our knowledge, the current study is the first to validate a Mandarin version for Chinese of the TFI. The factor analysis used to test the structural validity of the Chinese version of the TFI found it contained six factors EFA explored Chinese version TFI with fixed 8 factor analysis has 8 factors and consistent with the origin version, except the last factor 8 which only one item was loaded in. However, there are lots of limitation need to be further clarify, such as the sample size. Although the sample size determination for psychometric validation studies is always lack of clear scientifically sound recommendations on this topic [29]. In the present we recruited 206 tinnitus patients. And in the verification of the THI scale, this article compares the sample size of 199 cases of references in the Chinese version of THI [13], and the statistical results of the THI scale also agree with the results. Both THI and TFI have the same 25-items, the sample size should be also enough for the TFI, however the small size sample also could cause bias and may limited the validity and reliability of our findings. So, the bigger sample size should be recruited and be used to further validation. More and more literatures evaluate and validate new scales with both EFA and CFA [30, 31]. In the present we just used EFA to confirm the reliability and validity of the TFI-CN, we will collect more independent data to replicate and evaluate the construct validity of the scales with CFA. But we run omega hierarchical subscale to reconfirm the EFA validity, the results suggest TFI-CN has good reliability and validity. Finally, our study still demonstrated that the Mandarin version of the TFI for Chinese is a suitable instrument for measuring the impact of tinnitus. The reliability and validity of this version are very good and comparable with the original version of TFI. It can be used to assess tinnitus in clinical.

## Conclusion

The results suggest the Chinese versions of the TFI scales have high reliability and validity and can be used to assess the severity of clinical tinnitus and treatment-related changes in tinnitus. The TFI-CN has excellent internal reliability and satisfactory convergent and discriminant validity. Finally, one thing to emphasize is that having Chinese versions of tinnitus scales will make it possible for Chinese researchers to become active within international scientific networks. In light of the complex nature of tinnitus, collaboration between different research communities will be of great importance. By using the TFI-CN, Chinese scientists will not only be able to compare and report the results of their studies on tinnitus, but also become valued contributors on international scientific platforms.

## Data Availability

Please contact author for data requests.

## Abbreviations

TFI: Tinnitus Functional Index
THI: Tinnitus Handicap Inventory
CES-D: Center for Epidemiologic Studies-Depression Scale
BAI: Beck Anxiety Inventory
SWLS: Satisfaction with Life Scale
EFA: Exploratory factor analysis
THQ: Tinnitus Handicap Questionnaire
TAQ: Tinnitus Activity Questionnaire
TRQ: Tinnitus Reaction Questionnaire
TQ: Tinnitus Questionnaire
TFI-CN: Tinnitus Functional Index-China
THI-CN: Tinnitus Handicap Inventory-China
KMO: Kaiser-Meyer-Olkin
